# Pan-primate studies of age and sex

**DOI:** 10.1007/s11357-023-00878-3

**Published:** 2023-07-26

**Authors:** Steve Horvath, Amin Haghani, Joseph A. Zoller, Ake T. Lu, Jason Ernst, Matteo Pellegrini, Anna J. Jasinska, Julie A. Mattison, Adam B. Salmon, Ken Raj, Markus Horvath, Kimberly C. Paul, Beate R. Ritz, Todd R. Robeck, Maria Spriggs, Erin E. Ehmke, Susan Jenkins, Cun Li, Peter W. Nathanielsz

**Affiliations:** 1 Altos Labs, San Diego, CA USA; 2 Department of Biostatistics, Fielding School of Public Health, University of California, Los Angeles, Los Angeles, CA USA; 3 Department of Biological Chemistry, University of California, Los Angeles, Los Angeles, CA 90095 USA; 4 Department of Molecular, Cell and Developmental Biology, University of California Los Angeles, Los Angeles, CA 90095 USA; 5 Center for Neurobehavioral Genetics, Semel Institute for Neuroscience and Human Behavior, Department of Psychiatry and BiobehavioralSciences, David Geffen School of Medicine, University of California, Los Angeles, Los Angeles, CA USA; 6 Translational Gerontology Branch, National Institute On Aging Intramural Research Program, National Institutes of Health, Baltimore, MD USA; 7 The Sam and Ann Barshop Institute for Longevity and Aging Studies, and Department of Molecular Medicine, UT Health San Antonio, and the Geriatric Research Education and Clinical Center, South Texas Veterans Healthcare System, San Antonio, TX USA; 8 Altos Labs, Cambridge, UK; 9 Department of Neurology, David Geffen School of Medicine, University of California Los Angeles, Los Angeles, CA 90095 USA; 10 Department of Epidemiology, UCLA Fielding School of Public Health, University of California Los Angeles, Los Angeles, CA 90095 USA; 11 Corporate Zoological Operations, SeaWorld Parks, Orlando, FL USA; 12 Busch Gardens Tampa, SeaWorld Parks, Tampa, FL 33612 USA; 13 Duke Lemur Center, Durham, NC 27705 USA; 14 Texas Pregnancy & Life-Course Health Center, Southwest National Primate Research Center, San Antonio, TX USA; 15 Department of Animal Science, College of Agriculture and Natural Resources Department, Laramie, WY USA

**Keywords:** Primate, Baboon, Lemur, Strepsirrhine, Development, Epigenetic clock, DNA methylation

## Abstract

Age and sex have a profound effect on cytosine methylation levels in humans and many other species. Here we analyzed DNA methylation profiles of 2400 tissues derived from 37 primate species including 11 haplorhine species (baboons, marmosets, vervets, rhesus macaque, chimpanzees, gorillas, orangutan, humans) and 26 strepsirrhine species (suborders Lemuriformes and Lorisiformes). From these we present here, pan-primate epigenetic clocks which are highly accurate for all primates including humans (age correlation R = 0.98). We also carried out in-depth analysis of baboon DNA methylation profiles and generated five epigenetic clocks for baboons (Olive-yellow baboon hybrid), one of which, the pan-tissue epigenetic clock, was trained on seven tissue types (fetal cerebral cortex, adult cerebral cortex, cerebellum, adipose, heart, liver, and skeletal muscle) with ages ranging from late fetal life to 22.8 years of age. Using the primate data, we characterize the effect of age and sex on individual cytosines in highly conserved regions. We identify 11 sex-related CpGs on autosomes near genes (*POU3F2, CDYL, MYCL, FBXL4, ZC3H10, ZXDC, RRAS, FAM217A, RBM39, GRIA2, UHRF2*). Low overlap can be observed between age- and sex-related CpGs. Overall, this study advances our understanding of conserved age- and sex-related epigenetic changes in primates, and provides biomarkers of aging for all primates.

**Supplementary Information:**

The online version contains supplementary material available at 10.1007/s11357-023-00878-3.

## Introduction

During development, germline DNA methylation is erased, and then re-established in tissue-specific patterns as the developmental program unfolds after implantation [1]. The primary role of DNA methylation is to regulate the expression of genes, ensuring the production of appropriate ones in different tissues and organs. Our understanding of age-related changes in cytosine methylation has progressed rapidly [2–4] because of the technical advancement of methylation array platforms which simultaneously quantify thousands of individual CpGs at known locations on the human genome. Crucially, the correlation between methylation changes of some CpGs with chronological age over the course of an entire lifespan was found to be very strong [4–6]. This allowed to combine methylation levels of several CpGs and develop accurate age estimators, reviewed in [5–8]. An example is the human pan-tissue epigenetic age estimator which combines the weighted methylation average of 353 CpGs into an age estimate that is referred to as DNAm age or epigenetic age [9]. The discrepancy between epigenetic age and chronological age, termed epigenetic age acceleration, is associated with mortality risk and a wide range of age-related conditions in humans [10, 11]. This implies that epigenetic clocks successfully capture biological age, if not in its entirety, then at least to some measurable level, and offer measurement tools for anti-aging studies.

The original publication of the human pan tissue clock in 2013 reported a remarkable property of the human pan tissue clock: it also applied to chimpanzees [9]. But it was unknown whether one could build a pan tissue clock that applies to *all* primates. From a conceptual point of view, it would be desirable to have a pan-primate clock for measuring aging from conception to old age since it would satisfy the intuitive requirement that a molecular biomarker of aging should apply to all tissues from all primate species across the entire life course.

Here we present epigenetic clocks that accurately measure age in 37 primate species including strepsirrhines and haplorhines that diverged shortly after the emergence of the first true primates. To achieve this goal, we leveraged a mammalian methylation array platform that accurately measures cytosines in highly conserved regions of the DNA [12].

The current article addressed several goals: i) to develop epigenetic clocks that apply to all primate species, ii) to develop epigenetic clocks for baboons (olive-yellow baboon hybrids) which are promising animal models for anti-aging research, iii) to evaluate the effect of age on individual cytosine methylation levels in different primate tissues collected across their respective lifespans (EWAS of age), iv) to evaluate the effect of sex on individual cytosines (EWAS of sex) and v) to develop epigenetic estimators of sex that apply to all primates. We identify CpGs located on autosomes that relate to sex in most primate tissues.

In previous publications, we have presented epigenetic clocks for the vervet monkey [13], rhesus macaque [14], and common marmosets [15], and even universal pan mammalian clocks for all mammalian species [16]. However pan mammalian clocks are less accurate than clocks that are tailored for primates.

To develop pan-primate clocks, here we added novel data from baboons, lemurs and great apes. For ethical and practical reasons, anti-aging studies are difficult to conduct in great apes. The olive-yellow hybrid baboons living in the Southwest National Primate Research Center (SNPRC) can be employed to study biological changes including age-related processes [17]. Although baboons are an attractive primate model for studying aging, their lifespan, while considerably shorter than that of humans (max. 122.5 years), is still substantial (max. 37.5 years) [18], and leads to high maintenance costs, especially for studies on anti-aging interventions that use longevity as the primary measure of effect. The presented baboon clocks promise to greatly shorten the duration of baboon studies and hence lower the burden and costs associated with evaluating anti-aging interventions in this important primate species.

## Results

We generated DNA methylation data from *n* = 2398 tissue samples from 37 different primate species (Supplementary Table S1) including 26 strepsirrhine primate species (suborders Lemuriformes and Lorisiformes). For baboons specifically, we profiled *n* = 326 baboon tissues (*n* = 105 cerebral cortex samples, *n* = 48 heart, *n* = 41 adipose, *n* = 38 cerebellum, *n* = 50 liver, and *n* = 44 skeletal muscle, Supplementary Table S2). Unsupervised hierarchical clustering of the baboon samples reveals that the samples cluster largely by tissue type (Supplementary Fig. S1).

To facilitate cross species comparisons, we used the mammalian methylation array (HorvathMammalMethylChip40) that applies to all mammalian species [12]. While all of the 37492 CpG sites on the array can be mapped to the human genome, not all of these CpGs can be mapped to other primates. Out of 37492 CpGs on the array, 34865 could be aligned to specific loci that are proximal to 5781 genes in the Olive baboon (Papio anubis.Panu_3.0.100 genome), 36727 CpGs mapped to the vervet monkey (ChlSab1.1.100 genome), 35815 CpGs mapped to common marmosets (ASM275486v1.100 genome), 36733 CpGs mapped to rhesus macaques (Mmul_10.100), 31776 CpGs mapped to the gray mouse lemur (Microcebus_murinus.Mmur_3.0.100). Counts of CpGs for other primate species are presented in Supplementary Fig. S2A. A Venn diagram analysis reveals that 25727 CpGs on the array map to more than 10 primate species (Supplementary Fig. S2B).

### Epigenetic clocks for baboons

**Fig. 1:**
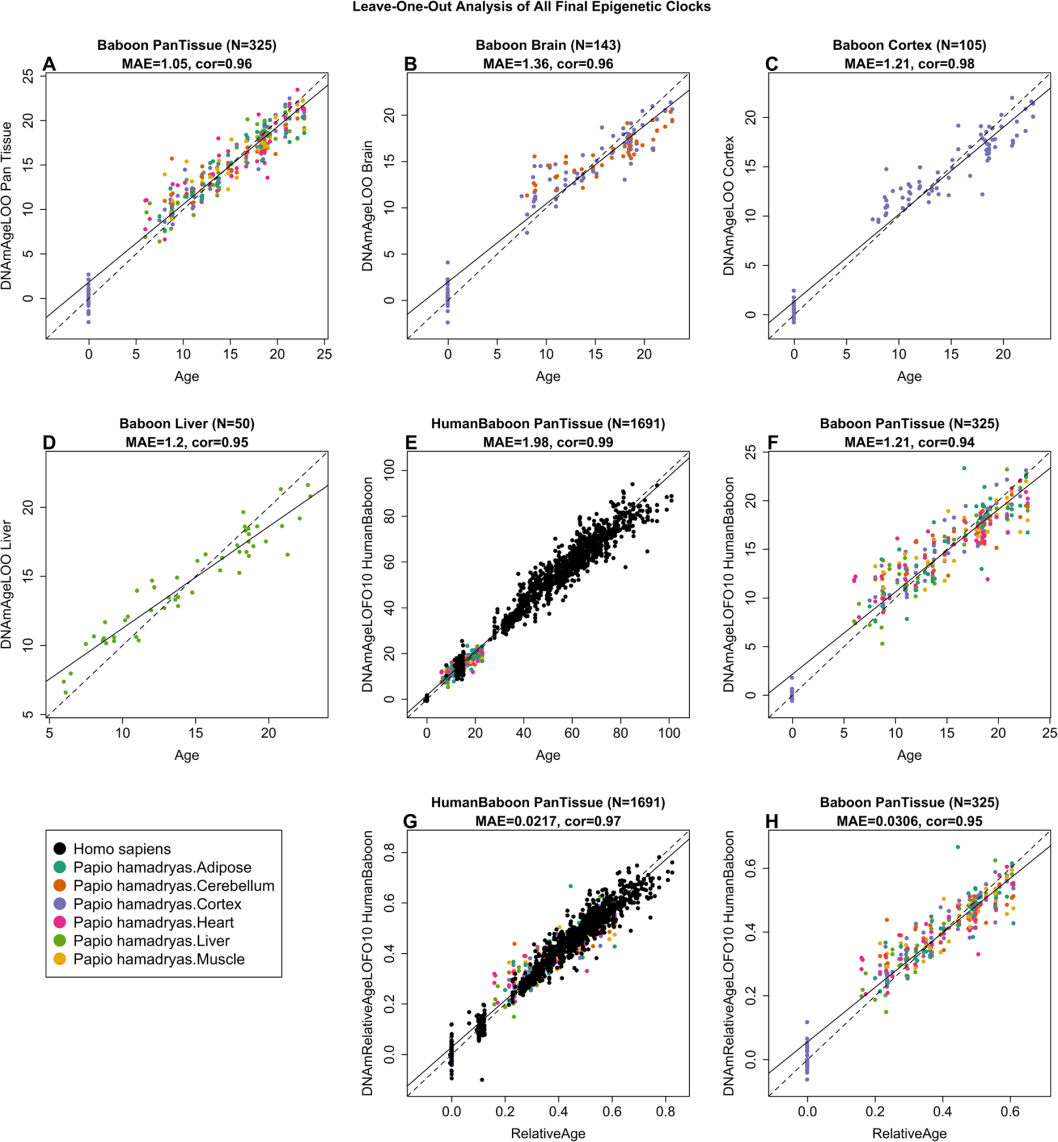
Cross-validation study of epigenetic clocks for baboons and humans. **A**-**D**) Four epigenetic clocks that were trained on baboon tissues: **A**) all tissues, **B**) all brain tissues, **C**) cerebral cortex, **D**) liver. **E**–**F**) Human-baboon clock for age applied to **E**) both species and **F**) baboons only. **E**) Human samples are colored in (black) and baboon samples (colored by tissue type). **G**, **H**) Human-baboon clock for relative age applied to **G**) both species and **H**) baboons only. Relative age was defined as the ratio of chronological age to maximum lifespan. Each panel reports the sample size, correlation coefficient, median absolute error (MAE). Dots are colored by tissue type or species. **A**-**D**) "LOO" denotes the leave-one-out cross validation estimates of DNA methylation age (y-axis, in units of years). **E**–**H**) "LOFO10" denotes the ten-fold cross-validation estimates of age (y-axis, in years)

To arrive at unbiased estimates of the epigenetic clocks, we carried out cross-validation analyses of the training datasets. This generates unbiased estimates of the age correlation R (defined as Pearson correlation between the estimated age (DNAm age) and chronological age) as well as the median absolute error, which indicates concordance of the estimated age with chronological age. The baboon epigenetic clocks that we constructed differed from each other with regards to tissue type, measure of age, and species (baboon only versus dual species human-baboon clock). Some clocks apply to all tissues (pan-tissue clocks, Fig. 1A) while others apply to specific tissues/organs and are named accordingly. We developed two brain-specific epigenetic clocks for baboons: one was trained using two brain regions (cerebellum and cerebral cortex, R = 0.96, Fig. 1B, Supplementary Fig. S3) and the other was only trained on cerebral cortex samples (R = 0.97, Fig. 1C). The baboon pan-tissue clock, which was trained on all available tissues, is highly accurate in age estimation across all different baboon tissue samples (R = 0.96 and median error 1.1 years, Fig. 1A, Supplementary Fig. S4), including cerebellum (R = 0.93, Supplementary Fig. S4C) and cerebral cortex samples (R = 0.99, Supplementary Fig. S4D). Although all of these different clocks exhibit high age correlations, pan tissue clocks may have different biological properties from tissue specific clocks, e.g. pan-tissue clocks tend to be less correlated with changes in cellular composition of the tissue [6].

We profiled all available baboon tissues. A significant advantage of our study was that all the different tissue samples in the baboon research originated from the same animals. This facilitated our ability to draw correlations between the acceleration of epigenetic age in one tissue type and that in another. For our pan tissue clock for baboons, we observed relatively weak correlations between epigenetic age acceleration across various baboon tissues. The highest correlations were found between the baboon cerebral cortex and cerebellum (r = 0.44), the cerebral cortex and heart (r = 0.36), the cerebral cortex and muscle (r = 0.30), as well as between adipose tissue and the liver (r = 0.40). The weakest correlations were found between baboon heart and muscle (r = 0), heart and liver (r = -0.01), adipose and cerebellum (r = 0), adipose and cerebral cortex (r = 0.031), adipose and muscle (r = -0.021).

To generate hybrid human-baboon clocks, human DNA methylation profiles were added to the baboon DNA methylation profiles in the training dataset. From these, two human-baboon pan-tissue clocks were developed. The human-baboon clock for *age* estimates age in units of years (Fig. 1E, F). The cross-validation analysis indicates that the human-baboon clock for age is highly correlated with age across both tissues from both species (R = 0.99, Fig. 1E) and across baboon tissues (R = 0.94, Fig. 1F).

The human-baboon clock for *relative age* estimates the ratio of chronological age to maximum lifespan of the species, with values between 0 and 1. This ratio allows alignment and biologically meaningful comparison between species with different average and maximal lifespans (baboon, 37.5 years and human, 122.5 years), which is not afforded by mere measurement of absolute age. The human-baboon clock for relative age is highly accurate when applied to both species together (R = 0.97, Fig. 1G) and only marginally less so when the analysis is restricted to baboon tissues (R = 0.95, Fig. 1H).

To determine the cross-tissue performance and the cross-species applicability of the baboon clocks, we applied them to an array of tissues from key human organs (*n* = 1352 from 16 human tissues). The application of pan-tissue baboon clock to *human* tissues produced epigenetic age estimates that are poorly calibrated; meaning that they exhibited poor concordance with chronological age (high median absolute error), but they nevertheless showed low to moderate correlations with ages in several human tissues (e.g. R = 0.49 in human adipose, R = 0.63 in human blood, R = 0.33 in human bone marrow, R = 0.76 in heart, R = 0.69 in kidney, R = 0.54 in lung, R = 0.44 in skeletal muscle, R = 0.93 in skin, and R = 0.54 in spleen, Supplementary Fig. S5).

### Primate clocks

We developed two pan-primate clocks that are expected to apply to all primate species. Toward this end, we used all available primate tissues (Supplementary Table S1). The two primate clocks estimate chronological age and relative age, respectively. Relative age is defined as the ratio of chronological age to the respective maximum lifespan of the species (Supplementary Table S3) based on an updated version of the "anAge" data base [19].

To evaluate the primate clocks, we used a 10-fold cross validation scheme that was balanced with respect to species, i.e. each fold contained the same proportion of species. We found that a square root transformation of age, more precisely sqrt(Age + 1), led to superior performance compared to the more widely used logarithmic transformation or identity transformation. The offset term of 1 year in the age transformation ensures that the clock is applicable to fetal samples as well (which are coded using negative age values). In terms of age transformations, our approach for constructing epigenetic clocks frequently utilizes the log linear age as seen in the human pan-tissue clock [9]. Other researchers have preferred the log transformation. However, our findings indicated that the square root transformation slightly outperformed both the log and log linear transformations, as evidenced by the cross-validation estimate of the R-squared measure. Despite the mathematical superiority of the square root transformation, its biological interpretability may be less straightforward.

**Fig. 2:**
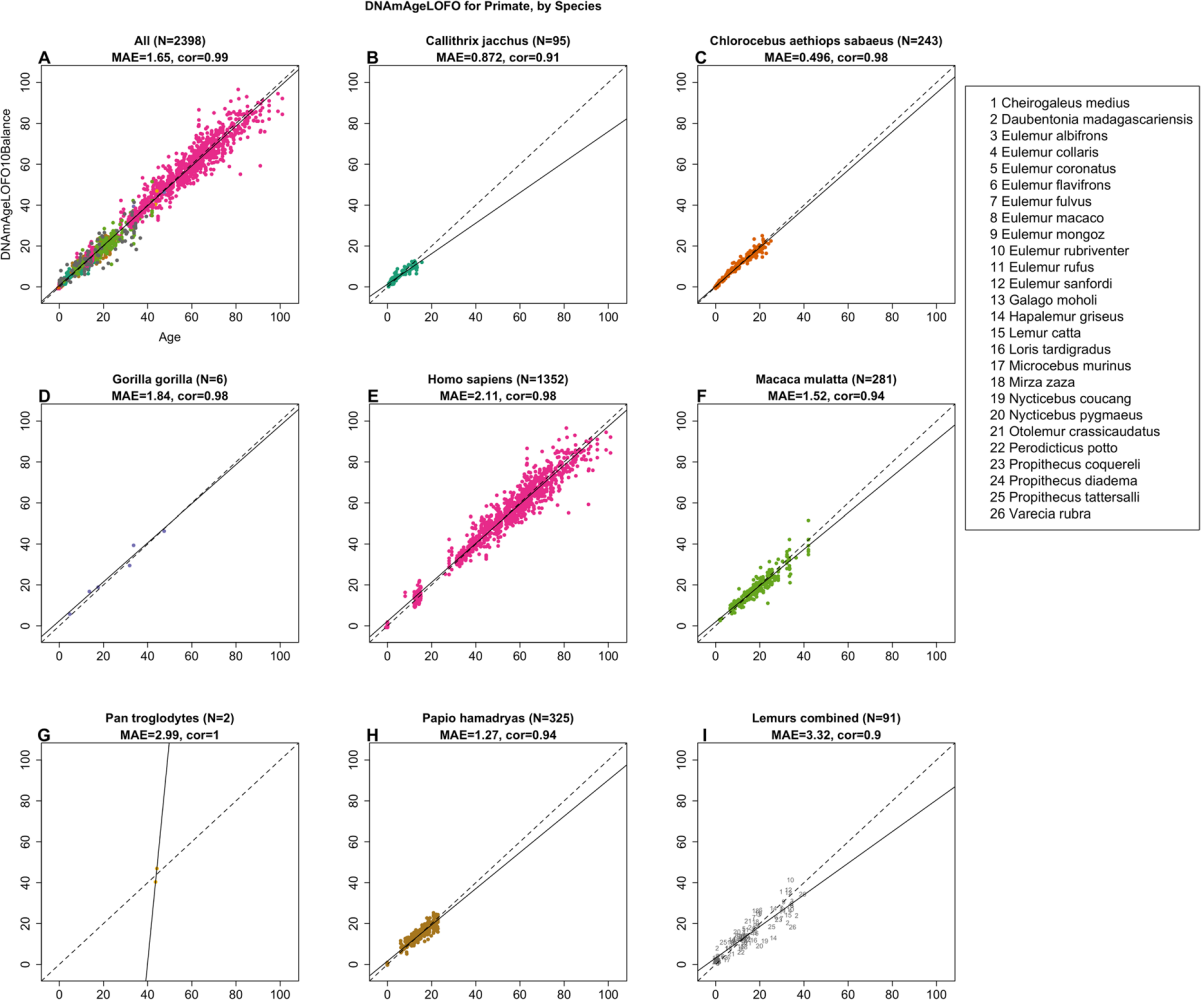
Cross-validation study of the primate clock for chronological age. **A**) Epigenetic clock based on tissues from all primate species (colored by species as indicated in the other panels). **B**-**I**) are excerpts from panel A but restricted to specific species mentioned in the title. **I**) Samples (dots) are blood and skin samples from 26 species of strepsirrhines as indicated in legend. Chronological age (years, x-axis) versus the ten-fold cross validation (balanced by species) of age (y-axis). Each panel reports the sample size, correlation coefficient, median absolute error (MAE). Dots are colored by species. The primate clock was developed by regressing sqrt(Age + 1) on cytosines that map to baboons and humans. We also developed a primate clock for relative age, defined as the ratio of age by the respective maximum lifespan (Supplementary Fig. S9). Details can be found in the Supplement

The primate clock for chronological age was highly accurate (R = 0.99, Fig. 2A) across all 37 primate species with an age correlation exceeding 0.90 in each species (Fig. 2B-F). The lowest accuracy was observed for blood and skin samples from strepsirrhini species (R = 0.90, Fig. 2I), which probably reflects that the underlying data come from 26 strepsirrhini species that diverged many millions of years ago, e.g., *Varecia rubra* diverged from *Eulemur fulvus* about 24.8 million years ago [20]. The primate clock for age also performs well with humans (R = 0.98, median error = 2.11 years, Fig. 2E) and compares favorably to the most accurate human clocks [21]. The primate clock for *relative* age is less accurate than that for chronological age (R = 0.96 versus R = 0.99, Supplementary Fig. S6) but it may be attractive for specific applications that want to adjust for the substantial differences in maximum lifespan. Details on the CpGs underlying the clocks can be found in Supplementary Table S4.

### EWAS of age in baboons

**Fig. 3:**
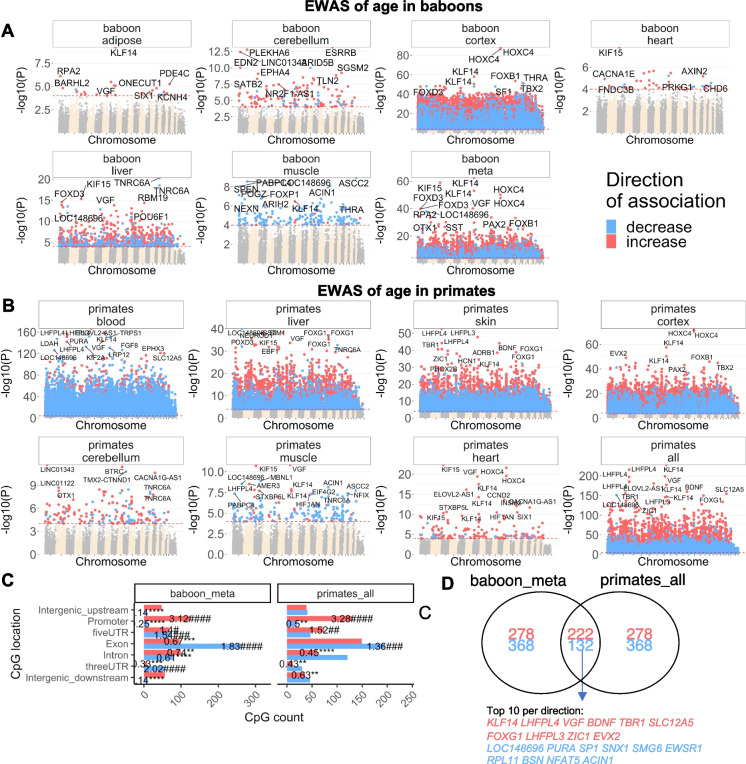
EWAS of age in baboons and primates. Meta-analysis of correlation tests in. **A**) Manhattan plots of the EWAS of chronological age in different baboon tissues (adipose, cerebellum, cerebral cortex, heart, liver, muscle of olive baboons). Note that the cerebral cortex leads to more significant *p* values (-log base 10 transformation of the p value, y-values) which reflects the broader age range of this tissue. **B**) Manhattan plots of the EWAS of chronological age primates. The p-values are based on Stouffer Z score meta-analysis of correlation with age in different tissues of primate species. “Primate-all” group is the Stouffer meta-analysis of age across all tissue types. The genome coordinates are estimated based on the alignment of Mammalian array probes to the Human Hg19 assembly. The direction of associations with *p* < 10^–4^ (red dotted line) is color-coded in red (age related increase in methylation) or blue (age related decrease). The top 15 CpGs were labeled by neighboring genes. **C**) Location of top CpGs (up to 500 per direction at *p* < 10^–4^ significance) in each tissue relative to the closest transcriptional start site. Significant odds ratios (count versus background) are reported for each bar. Fisher exact *p* values are encoded as follows *,# *p* < 0.05, **,## *p* < 0.01, ***,###*p* < 0.001, ****,####*p* < 0.0001. **D**) Venn diagram of the overlap of the meta-analysis of age in baboons and primates

To study tissue and species-specific aging effects on cytosine levels, we carried out an Epigenome-Wide Association study (EWAS) of age in different baboon tissues (Fig. 3A). The EWAS results for other primate species can be found in our companion papers [14, 15] (Methods).

The creation of a pan-tissue clock that can gauge age across all tissues might erroneously imply that the influence of age on cytosine methylation levels is significantly conserved across various tissues. Yet, this is not the reality; it's actually quite the opposite. This is emphasized by the weak correlation between EWAS of age results in one baboon tissue compared to another (see Supplementary Fig. S7). The scant conservation of aging effects across different tissue types might be due to the limited sample size of non-cortex tissues (see Supplementary Table S2). However, a similarly low conservation of EWAS results has been noted in human studies too [22]. The disparities in EWAS results across tissues do not stem from using different animals; indeed, all the tissues examined were derived from the same baboons. To sum up, while age does have a tissue-specific effect on cytosine methylation levels, the successful design of pan-tissue clocks suggests that specific CpGs, especially those located in the binding regions of the polycomb repressive complex 2, prove instrumental in building age estimators applicable across various tissues.

To capture the most-affected loci in all baboon tissues, a nominal p value < 10^–4^ was employed. Apart from *p* values, we also report correlation test Z statistics, which follow a standard normal distribution under the null hypothesis of zero correlation. Positive and negative values of the Z statistics correspond to age related gain/loss of methylation. The top age related CpGs in specific baboon tissues were located in the following genes: *KLF14* promoter (Z = 6.2, adipose); *ESRRB* intron (Z = 7.5, cerebellum); *HOXC4* promoter (Z = 19.9, cerebral cortex); *KIF15* promoter (Z = 5.6, heart); *TNRC6A* exon (Z = -9.4, liver); and *ASCC2* exon (Z = -6.1, muscle). To identify age-associated CpGs that are shared across the tissues, we carried out Stouffer's meta-analysis across the six tissue types. Meta-analysis across baboon tissues implicated *KLF14* (Z = 16.7)*, KIF15* (Z = 16.3), and *HOXC4* (Z = 15). In addition, two CpGs in the exon of *FOXD3* (Z = 13.3) exhibit positive correlations with age (Supplementary Table S5). Not all of these CpGs correlate with age in strepsirrhines as shown in the following.

### EWAS of age in all primates

For seven different tissue types (blood, cerebellum, cerebral cortex, heart, liver, skeletal muscle, skin), we carried out a meta-analysis EWAS of age across primate species (Fig. 3B). We observe tissue specific aging patterns (Supplementary Fig. S7). For example, the top EWAS hits in primate blood (*LHFPL4, KLF14*) are not among the top hits in primate liver (Fig. 3B). When focusing on blood samples, *ELOVL2-AS1* is particularly noteworthy since *ELOVL2* has been implicated in human epidemiological studies [4, 23]. Cytosine methylation levels in *ELOVL2* appear to regulate aging in the retina [24].

To identify CpGs that relate to age in most tissues and primates, we used a two-step meta-analysis approach to combine the EWAS results of age across 29 strata (defined by primate species and tissue type for which at least 10 samples were available). We find that CpGs near *KLF14* and *LHFPL4* gain methylation in most primate tissues (Supplementary Table S6). A CpG near *ELOVL2-AS1* (cg16867657) correlates positively with age in several species (*Chlorocebus aethiops sabaeus*, *Homo sapiens*, *Macaca mulatta*, and *Papio anubis)* but not in *Callithrix jacchus* or strepsirrhines (Supplementary Table S6). A CpG (cg14361627) underlying *KLF14* is significantly correlated with age in all Haplorhine species but not in strepsirrhines (Supplementary Table S6).

Many CpGs correlate *positively* with age in all considered primate species (both haplorhines and strepsirrhines) including three CpGs near *LHFPL4* (cg12841266, cg24866418, cg11084334) and CpGs near *LHFPL3*, *BDNF, TBR1, SLC12A5, FOXG1* (Supplementary Table S6)*.*

Conversely, we find CpGs that correlate *negatively* with age in all primate species including CpGs near *TRPS1 SNX1, SMG6, ARID5B, EWSR1* (Supplementary Table S6)*.* However, we caution the reader that CpGs near these genes can have tissue specific aging patterns.

There is strong agreement between our EWAS findings in baboons and those across all primates. Out of the top 500 CpGs with a positive age correlation in baboon tissues, 222 overlap with the top 500 CpGs with a positive age correlation across all primate tissues (Fig. 3D). A lower overlap (132 out of 500 CpGs) between baboons and primates can be observed for CpGs with negative age correlations. Overall, we find that CpGs with negative age correlations exhibit lower conservation across tissue type and primate species (Fig. 3D).

Age-related CpGs map to both genic as well as intergenic regions, which are defined relative to transcriptional start and stop sites (Fig. 3C). However, the relationship to age seems to depend on the relative gene region of the CpG. CpGs in promoters tend to gain methylation with age in primates, including baboon samples (odds ratio > 3.3, *p* < 10^–4^; Fig. 3C). In contrast, the CpGs in exon regions tend to lose methylation with age (odds ratio > 1.3, *p* < 0.01; Fig. 3C).

**Fig. 4:**
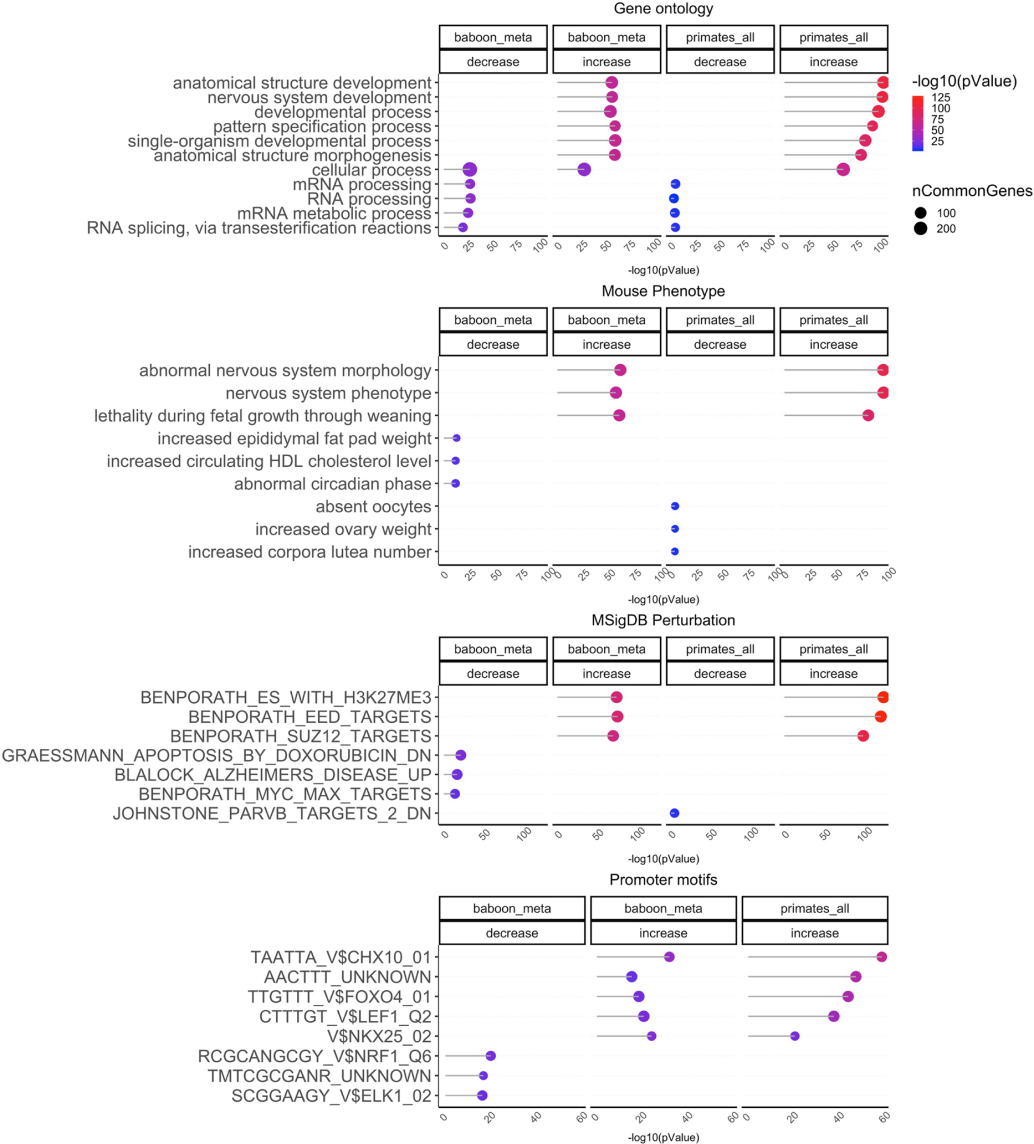
Gene set enrichment analysis of aging effects in primates. The genome region based enrichment analysis was carried using GREAT [25] where the statistical background was defined by the probes on the mammalian methylation array in Hg19 assembly. The CpGs are annotated with adjacent genes with gene regulation domain: 5.0 kb upstream, 1.0 kb downstream, plus Distal: up to 50 kb. We extracted up to top 500 CpGs based on p value of association per direction of change as input for the enrichment analysis. The *p* values are calculated by genomic region based hypergeometric test with the following ontologies: gene ontology, mouse phenotypes, promoter motifs, and MsigDB Perturbation, which includes the expression signatures of genetic perturbations curated in GSEA database. The results were filtered for significance at *p* < 10^–5^ and only the top terms for each EWAS are reported

Enrichment studies show that CpGs with positive age correlations tend to be located i) in regions targeted by Polycomb Repressive Complex 2 (characterized by histone mark H3K27ME3) in embryonic stem cells, ii) near genes that play a role in development, and iii) in regions bound by transcription factors CHX10 and FOXO4 (Fig. 4). CpGs with negative age correlations are located near genes that play a role in mRNA processing (Fig. 4, Supplementary Table S7).

### Sex effects in primates

We aimed to find CpGs that relate to sex in many different tissues and primate species. However, we excluded the common marmoset from our analysis, because marmosets appear to have a unique characteristic among mammals tested when it comes to DNA methylation studies: it is not possible to predict sex on the basis of methylation data in common marmosets despite development of clocks that quite accurately predict age [15]. As discussed in [15], this probably reflects that the blood from marmosets is chimeric. Most marmosets carry a proportion of blood cells that partially reflect the germline of their dizygotic co-twin [26]. Since this process occurs with opposite sex dizygotic twins, female marmosets can carry blood cells derived from their brothers (with Y-chromosomes) and vice versa.

We used the data from all other primates to identify sex-related CpGs in multiple tissues. For each species, we focused on the subset of CpGs that map to the respective genome (e.g. 37492 CpGs for humans, 34939 for vervet monkey, 34865 for baboons). To find sex related CpGs, we regressed cytosine methylation (dependent variable) on both age and sex. In total, the analysis involved 36 regression models in 13 tissues from human, baboon, vervet monkey, macaque, and strepshirrhine primates from prenatal time to late adulthood.

**Fig. 5:**
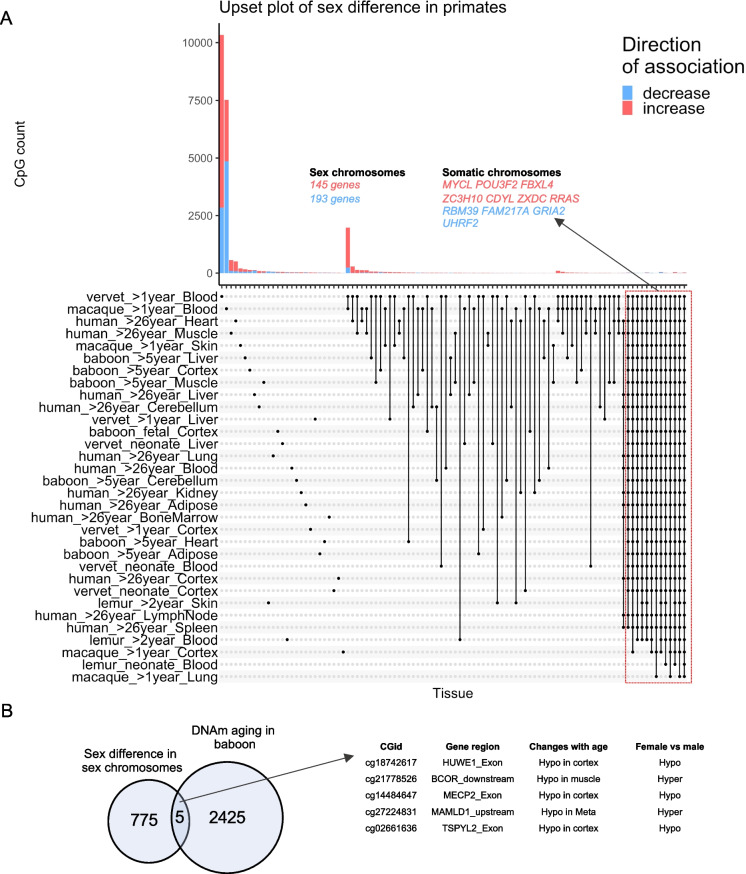
Sex effects on DNA methylation in primates. **A**) The upset plot of the sex-mediated differential methylated CpGs in 13 tissues from 30 primate species (human, baboon, vervet monkey, macaque, and 26 strepsirrhine primate species which where were analyzed together and denoted as lemur) at different age ranges. Red (blue) color codes genes/CpGs with significantly increased (decreased) methylation levels in females compared to males. The analysis included 30 multivariate regression models (stratified by species, tissues, and age categories), including chronological age as the covariate. The y-axis reports the number of CpGs that are significant at a 5% FDR rate. Neonates in vervet monkeys and strepsirrhine primates were aged < 1 year. 780 CpGs adjacent to 248 genes related to sex in > 19 of these models. **B**) The overlap of conserved sex-dependent CpGs and DNAm aging in the baboons. Manhattan plots of the sex differences in specific baboon tissues can be found in Supplementary Fig. S10. The box plot of sex differences in these CpGs are reported in Supplementary Fig. S11

The median number of sex-associated methylation positions (SMP) in all these models was 1117 CpGs (at a 5% False Discovery Rate), most of which were located on the X chromosome. The largest number of SMPs (more than 7000 CpGs) could be found in blood samples from macaque and vervets older than age 1 (Fig. 5A). By contrast, we found only 1022 SMPs in human blood. In humans, the heart and muscle samples had a higher number of SMPs (2497 and 2226 CpGs, respectively). In specific tissues (heart and muscle in humans, blood in macaque and vervet monkey), we find that a surprisingly large number of SMPs are located on *autosomes* even though in most tissues, the number of autosomal SMPs is very limited (fewer than 200 in most tissues). In total, 780 SMPs were significantly associated with sex in more than 19 out of 36 regression models and 25 X chromosomal CpGs are related to sex in 32 models (Supplementary Table S8).

As expected, most of the sex-related CpGs, namely 769 CpGs, are located on sex chromosomes reflecting X chromosomal inactivation in females. The 11 autosomal sex-related CpGs (1.3%) are proximal to 11 genes (Fig. 5A, Supplementary Table S8). Three of these genes (*FAM217A, CDYL, POU3F2*) are located in human chromosome 6 while the remaining autosomal sex-related CpGs are located near *MYCL, ZC3H10, ZXDC, RRAS, RBM39, GRIA2, UHRF2,* and *FBXL4* (Fig. 5A).

Of the 780 SMPs, only 5 CpGs correlate significantly with age in baboons (CpGs near *HUWE1*, *BCOR, MECP2, MAMLD1, TSPYL2,* Fig. 5B).

Detailed enrichment analysis of sex-differences in all primates can be found in Supplementary Fig. S8 and Supplementary Table S9. As expected, enrichment analysis of the sex-associated CpGs highlighted gonosomal and X-linked inheritance for CpGs located on both sex or autosomal chromosomes in all primates. The genes on sex chromosomes were also related to testicular development and several cognitive and neuronal pathways including intellectual disability, cognitive impairment, autism spectrum disorder, mental health, agitation, and thermal nociceptive threshold. Strikingly, the genes on the autosomal chromosome were also enriched in several neuronal pathways such as GABA receptor activation, intellectual disability, and synaptic function. Thus, our result suggests a possible difference in neuronal development, activity or function between sexes across these primate species that are associated with epigenetic changes. These differences are apparent from the fetal stage to very old age.

### Sex effects in baboons

We used the baboon samples to provide a detailed analysis of sex effects in different tissues and age groups. These results largely replicate the above-mentioned findings for all primates. For example, the same 11 autosomal locations continue to be associated with sex (Supplementary Fig. S9, Supplementary Table S8). The GREAT enrichment analysis in baboons leads to similar enrichments including X-linked inheritance and intellectual disability (Supplementary Fig. S10).

### Sex predictors in all primates

Methylation based sex predictors can be useful for finding platemap errors and confirming identity of expected donor samples. Using elastic net regression, we developed a multivariate predictor of sex that applies to all primates except for marmosets (Supplementary Table S10). The predictor of female sex is based on 86 CpGs that are located on the X chromosome. A cross validation analysis suggests that the predictor is 99% accurate, e.g. it misclassified a single human sample (out of *n* = 1352 samples). We also carried out an EWAS of sex that ignored species and tissue type. Across all primate samples from 37 primate species, 294 X-chromosomal CpGs markers were significantly hypomethylated and 165 CpGs were hypermethylated in females at a significance threshold of *p* = 10^–300^ (Supplementary Table S11). Boxplots for six representative CpGs are presented in Supplementary Fig. S11.

### Sex effects in large human cohorts

To test whether the sex-specific results in primates could be corroborated in human methylation data generated on another genomic platform, we analyzed human Illumina 450 k array data from three large studies: blood samples from individuals of European ancestry (Framingham Heart study), blood samples from individuals of African ancestry (Jackson Heart study), and postmortem prefrontal cortex samples from individuals from the Religious Order study (ROSMAP). CpGs can fall in several regions relative to the transcriptional start site of a gene e.g. the gene promoter, gene exon, 5' untranslated regions, 3' UTR, intergenic regions.

**Fig. 6:**
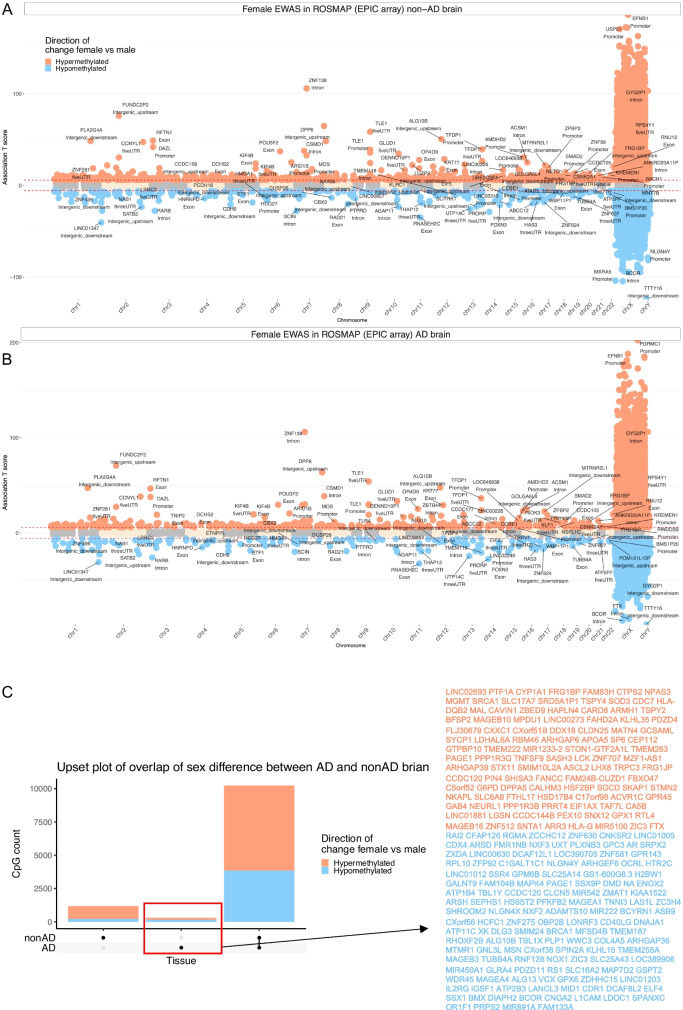
Sex related methylation changes in primates implicated by two array platforms. The Manhattan plots only involves EPIC array probes that map to the same human gene regions as the corresponding probe on the mammalian array. The y-axis reports sex effects (Z statistics) based on the human EPIC array. The x-axis reports genomic locations (gene regions) that were significant (5% FDR) in at least one of the primates’ blood samples (human, macaque, vervet monkey). **A**) The overlap of primates EWAS of sex with the meta-analysis of blood samples from the Jackson Heart (JHS) and Framingham Heart (FHS) Studies. Red (blue) color codes genes/CpGs with significantly increased (decreased) methylation levels in females compared to males. The meta-analysis z score is calculated by Stouffer's method from the EWAS of sex (adjusted for age) in JHS and FHS. The red lines are z scores that corresponds to *p* < 10^–100^ in EPIC array meta-analysis. The top two gene regions from each chromosome were labeled. **B**) The overlap of primates EWAS of sex (adjusted for age) in prefrontal cortex samples from ROSMAP. The probes were also further filtered for the gene regions that were significant (5% FDR) in at least one of the primates’ cerebral cortex samples (baboon, vervet monkey). The red lines are Student T test statistics that corresponds to a significance threshold of *p* < 10^–8^ in the EWAS analysis. The human EPIC array covers most of the gene regions that are covered by the mammalian methylation array (Supplementary Fig. S12)

Since the mammalian array and the human 450 k array share 5241 CpGs [12], we compared the results of the two platforms at the level of gene region. Almost all human gene-regions that contain at least one CpG from the mammalian array are also covered by the human 450 k array (Supplementary Fig. S12). Several gene-regions proximal to these SMPs in primates were also differentially methylated between human males and females in both blood and brain samples (Fig. 6). Interestingly, this analysis also highlighted autosomal gene *POU3F2* on chromosome 6, which was one of the 11 autosomal sex-related genes across primates. Significant sex-related CpGs in human brain samples are reported in Supplementary Fig. S13.

## Discussion

The original paper on the human pan tissue clock provided limited evidence that one could build epigenetic clocks that apply to several primates species [9]. But the rigorous testing of this hypothesis required two advances. First, a robust measurement platform that applies to highly conserved stretches of DNA [12]. Second, a large collection of tissues from many primate species. The *n* = 2400 tissue samples from 37 primate species cover large evolutionary distances between primates. The island home of lemurs (Madagascar) separated from Africa about 145 million years ago but molecular estimates based on mitochondrial genomes suggest that strepsirrhines (e.g. lemurs) diverged from haplorhines (e.g. humans) about 74 million years ago [20, 27]. Humans separated from the old-world monkey lineage approximately 23–25 million years ago, the baboon and rhesus macaque lines separated approximately 9 million years ago, and the baboon and vervet monkeys separated 10 million years ago [20].

Despite the evolutionary distance between the 37 primate species, we managed to construct two highly accurate primate clocks based on a single multivariate model. This effectively consolidates the notion that certain epigenetic aspects of aging are highly conserved between primate species. Our primate clock for age is more accurate than the clock for relative age (age correlation R = 0.99 vs R = 0.96, Fig. 2A, Supplementary Fig. S6). However, it was important to apply a square root transformation to age to achieve this high accuracy. The use of the square root transformation is a bit unusual in this context since logarithmic transformations are more common [9, 28, 29].

Our Mammalian Methylation Consortium has generated clocks for dozens of species and even specific taxonomic groups such as bats, marsupials [16, 30–57]. This work has culminated in the development of universal pan mammalian methylation clocks [16]. Although pan-mammalian clocks demonstrate high accuracy in primates [16], our data suggests that the introduced pan-primate clocks outperform them in terms of accuracy. Future investigations are required to determine which of these clocks best represents organismal health. This could include examining which clock reacts predictably to interventions that modify lifespan.

The ability to generate primate clocks should not detract from the fact that there are profound differences in age-related methylation changes across species and even between tissues from the same species. Indeed, when age-associated CpGs were analyzed, their tissue specificity was readily apparent (Supplementary Fig. S7). This can appear at first sight to be inconsistent with the successful development of a pan-tissue clock. It is however important to bear in mind that age-associated CpGs that constitute the clock are not necessarily the highest scoring ones for individual tissues. As such, it appears that as tissues age, two sets of CpGs undergo methylation change; the first is tissue-specific and the second is general across all tissues, which is consistent with what has been observed in humans [3, 58, 59].

A CpG in the promoter of *KLF14* was the top age-related CpG across all baboon tissues and also the top hit in our meta-analysis of primate tissues (Fig. 3) but this CpG does not correlate with age in the strepsirrhines (Supplementary Table S6). *KLF14*, is a transcription regulator that can activate and also repress the expression of genes. Although its direct targets remain to be fully characterized, its pathologic effect is known to include prevention of cardiovascular disease [60]. Methylation of *KLF14* locus in the blood of mice is predictive of chronic inflammation in adipose tissue [61], suggesting that this cross-tissue age-associated locus might extend to other tissues and may bear even greater predictive scope. Indeed, *KLF14* is also one of the top EWAS findings in primate blood. Human epidemiological studies have revealed that methylation of this locus in blood, saliva and buccal swabs is predictive of age in humans [62], and along with *ELOVL2*, *KLF14* is one of the most consistently identified loci with age-associated methylation change [63].

We identified many CpGs that showed a consistent aging pattern across all primate species (haplorhines and strepsirrhines) e.g. the positively age-related CpGs near *LHFPL4, LHFPL3, BDNF, TBR1, SLC12A5, FOXG1* and negatively age-related CpGs near *TRPS1, SNX1, SMG6, ARID5B, EWSR1* (Supplementary Table S6).

Our meta-analysis of age across baboons and primate tissues implicated CpGs near several members of Hox family genes (e.g. *HOXC4*, *HOXA11, HOXD8,* and *HOXC10)* which gain methylation with age. The Hox family is one of the oldest gene sets that are conserved across not only mammals but arthropods as well. Hox proteins specify development of body segments of organisms and are pivotal for normal healthy development. Their expression is regulated by polycomb repressive complex, whose DNA targets are consistently identified, as well in this study, as being increasingly methylated with age [3, 5]. Hence the involvement of HOX loci further implicates the importance of the developmental process in aging. This is further validated through the enrichment analyses of age-associated CpGs that were identified with developmental processes across the different tissues.

Apart from two primate clocks that apply to all primate species, we present five epigenetic clocks that are specific for baboons and are applicable to their entire life course (from birth to old age). The specific baboons used to develop the reported clocks herein are hybrid animals resulting from the mating between olive and yellow baboons. We could not evaluate whether methylation rates might be different in hybrids as compared to pure-bred members of one or the other type in this study. However, our experience with other hybrid species (e.g. peromyscus [64]) leads us to predict that any differences would be minor. In the worst case, the baboon clocks would be offset by a constant value between DNAmAge and actual age. By definition, our primate clock applies to all primate species and would certainly apply to different species of baboons. Another group has previously presented a blood clock for baboons based on DNA methylation profiles that were generated using a different methylation profiling platform (Reduced Representation Bisulfite Sequencing) [65]. We could not evaluate this blood clock in our data because the two genomic platforms profiled different sets of CpGs and furthermore, we did not profile blood samples in baboons.

Sex affects the blood methylome in a species and life stage dependent manner. In neonatal blood from vervets, we observed few autosomal CpGs that relate to sex but this number increases dramatically in older animals. While blood samples from older macaques and vervets also reveal a large number of autosomal CpGs that relate to sex, the same could not be observed in human blood samples. We present CpGs that relate to sex in all primate species. A subset of these CpGs allowed us to develop a multivariate estimator of sex that applies to all primate species and all tissue types. Sex predictors can be used to find plate map errors or other data handling errors. Most of the sex-related CpGs were located on the X chromosome. However, our EWAS of sex also identified 11 autosomal locations located near 11 genes (*POU3F2, CDYL, MYCL, FBXL4, ZC3H10, ZXDC, RRAS, FAM217A, RBM39, GRIA2, UHRF2*). None of these locations overlap with autosomal sex-associated co-methylated regions that have recently been described in humans [66]. However, we could confirm the *POU3F2* gene location in humans using large human data sets generated on the human 450 k array (Fig. 6). According to genecards (https://www.genecards.org/) six of the 11 genes play distinct sex related role as briefly described in the following. *MYCL* (Proto-Oncogene) plays a role in breast disease and other female related cancers. *FBXL4* is related to breast carcinoma. *ZC3H10* is overexpressed in the ovaries. *ZXDC* is overexpressed in the placenta. RRAS has been associated abnormality of male external genitalia (cryptorchidism). *CDYL* is a primate-specific Y-chromosomal gene family expressed exclusively in the testis but this autosomal gene is ubiquitously expressed. *CDYL* encodes a positive regulator of polycomb repressive complex 2 (PRC2) [67, 68]. This is particularly relevant as PRC2 target sites become increasingly methylated with age across different mammalian species [3, 39]. The collective evidence from many different species analyzed thus far repeatedly points to PRC2 as a pivotal factor underlying epigenetic aging. Therefore, the difference in methylation rate of the *CDYL* locus could potentially result in differences in PRC2 activity.

Beyond their utility, the primate and baboon epigenetic clocks reveal several salient features with regard to the biology of aging. First, the fact that these are pan-tissue clocks re-affirm the notion that aging is a coordinated biological process that is harmonized throughout the body. Second, the developmental process is further implicated as being an important component of the process of epigenetic aging. Third, the ability to develop human-baboon clocks and even primate clocks attests to the high conservation of the aging process across evolutionary distinct primate species. This implies that treatments that alter the epigenetic age of baboons, as measured using the human-baboon clock may be more likely to exert similar effects in humans than treatments developed from more distantly related species.

We expect that the availability of the baboon and primate clocks will provide a significant boost to the potential use of baboons and other primates as models for human aging.

## Methods

### Ethics

This research complied with all relevant ethical regulations overseen by seven ethics review boards. The human skin samples were acquired with informed consent prior to collection of human skin samples with approved by the Oxford Research Ethics Committee in the UK; reference 10/H0605/1. Participants were not compensated. The secondary use of the other de-identified/coded human tissue samples (blood, postmortem tissues) is not interpreted as human subjects research under U.S. Department of Health & Human Services 45 CFR 46. Therefore, the need to obtain written, informed consent from human study participants was waived (secondary use of de-identified tissues). Human samples were covered by University of California Los Angeles IRB#18-000315. All procedures related to non-human primates were approved by different committees: baboons (UTHSCSA Animal Care and Use Committee), strepsirrhini (Duke Institutional Animal Care and Use Committee and the DLC Research Committee), rhesus macaques (Animal Care and Use Committee of the NIA Intramural Program) [14], vervet monkey (UCLA and VA Institutional Animal Care and Use Committees) [13], marmosets (IACUC of UTHSA) [15].

#### Baboon care and maintenance

All animals were given a full veterinary examination prior to recruitment to the study and no obvious cause of ill health or pathology was observed. The animals were housed in group cages at the Southwest National Primate Research Center, at Texas Biomedical Research Institute (TBRI), in San Antonio, Texas in mixed sex groups of up to 16. The remaining 4 females were housed in individual cages at the UT Health Sciences Center San Antonio (UTHSCSA).

Twenty-eight females and the ten males were fed ad libitum Purina Monkey Diet 5038 (12% energy from fat, 0.29% from glucose and 0.32% from fructose and metabolizable energy content of 3.07 kcal/g protein; Purina LabDiets, St Louis, MO, USA) (CTR). Water was continuously available to all animals. Animal health status was recorded daily.

### Necropsy

None of the animals were euthanized for this project. Rather, we used left-over frozen tissue samples that had previously been collected as part of other projects. Necropsies were performed by either a qualified, experienced veterinarian or M.D investigator. At TRBI, baboons were pre-medicated with ketamine hydrochloride (10 mg/kg IM) and anesthetized using isoflurane (2%) resulting in general anesthesia as previously described [69]. Baboons were exsanguinated while under general anesthesia as approved by the American Veterinary Medical Association. At UTHSCSA four animals were euthanized using Pentobarbital at 390 mg/ml (Fatal- Plus Solution, Vortech, Dearborn, MI, USA). Following cardiac asystole, respiratory failure and a lack of reflexive response to both skin pinch and eye touch stimulation, tissues (adipose, cerebellum, cerebral cortex, muscle, heart, liver) were rapidly dissected and immediately frozen in liquid nitrogen.

For the studies in which fetal tissue was obtained, all animals were housed in 20 foot × 20 foot × 15 foot metal and concrete group cages at the Texas Biomedical Research Institute. Experimental animals were obtained from appropriate groups of 16 healthy female baboons of similar pre-study body weights (10–15 kg) and morphometric features (13). The potential day of conception was determined based on the day of ovulation and changes in sex skin color and pregnancy was confirmed at 30 days post ovulation by using ultrasonography. Details of housing, feeding, and environmental enrichment have been published elsewhere (13). All procedures were approved by the University of Texas Health Science Center and Texas Biomedical Research Institute internal animal care and use committees and performed in the Association for Assessment and Accreditation of Laboratory Animal Care–approved facilities.

Prior to Cesarean section, baboons were premedicated with ketamine hydrochloride (10 mg/kg, IM). Following tracheal intubation, isoflurane (2%, 2L/min, by inhalation) was used to maintain an appropriate plane of anesthesia throughout the surgery. A cesarean section was performed at gestational day 165 (0.9 of gestation) using standard sterile techniques as previously described [70]. Following hysterotomy, the umbilical cord was identified and used for fetal exsanguination with both maternal and fetal baboon under general anesthesia as approved by the American Veterinary Medical Association Panel on Euthanasia. Postoperatively, mothers were placed in individual cages and watched until they were upright under their own power. Maternal analgesia was administered for 3 days (buprenorphine hydrochloride injection; Hospira, Inc., Lake Forest, IL, USA; 0.015 mg/kg/day) post-operatively or longer if indicated. They were returned to their group cage two weeks postoperatively.

Animals were individually fed to enable precise regulation of intake either between 7:00 am and 9:00 am or 11:00 am and 1:00 pm as described in detail elsewhere [71]. Water was continuously available in each feeding cage (Lixit, Napa, California), and the animals were fed Purina Monkey Diet 5038 (Purina, St Louis, Missouri). For this study, we selected samples representing the entire primate lifespan, from neonate to old age (Supplementary Table S2).

#### Strepsirrhine primates

Strepsirrhini is a suborder of primates that includes the lemuriform primates, which consist of the lemurs of Madagascar, pottos and galagos from Africa, and the lorises from Southeast Asia. Lemuroids and lorisoids together form the more ancestral sister clade to all other living primates. As such, they lend unparalleled power to any comparative study within the primate clade.

For this study, we selected a total of 91 samples from individuals representing 26 strepsirrhine species, in most cases, the entire lifespan, from immature (infant or juvenile) to senile stages: 68 samples from peripheral blood, 23 samples from skin (Supplementary Table S1). The strepsirrhine primates (suborders Lemuriformes and Lorisiformes) used in this study were from the Duke Lemur Center (DLC) in Durham, NC (USA). The Duke Lemur Center is certified by both the Association for Assessment and Accreditation of Laboratory Animal Care and the American Zoological Association. The animal handling and sample collection procedures in this study were performed by a veterinarian after review and approval by the Duke Institutional Animal Care and Use Committee and the DLC Research Committee. Both housing and sample collection met or exceeded all standards of the Public Health Service's “Policy on the Humane Care and Use of Laboratory Animals”. The lemurs are housed in comparable social and housing conditions, habituated to human presence, and individually identifiable. The DLC also maintains a large collection of banked tissues, deriving from routine veterinary procedures and necropsies, amassed over the Center’s 55-year history. Detailed records of life and medical history, reproduction, and social-group membership are digitally maintained [72].

Peripheral blood was collected through venipuncture with standard procedures, either during a routine veterinary procedure or at time of necropsy. Skin tissues were collected during necropsies. Whole blood was preserved in either EDTA or Lithium Heparin, and stored at -80 oC. Skin tissues were either frozen directly at -80 oC or were first flash frozen and then stored at -80 oC.

We profiled the following species: Cheirogaleus medius (Fat-tailed dwarf lemur), Daubentonia madagascariensis (Aye-aye), Eulemur albifrons (White-headed lemur), Eulemur collaris (Collared brown lemur), Eulemur coronatus (Crowned lemur), Eulemur flavifrons (Blue-eyed black lemur), Eulemur fulvus (Brown lemur), Eulemur macaco (Black lemur), Eulemur mongoz (Mongoose lemur), Eulemur rubriventer (Red-bellied lemur), Eulemur rufus (Red-fronted lemur), Eulemur sanfordi (Sanford's brown lemur), Galago moholi(South African galago), Hapalemur griseus(Bamboo lemur), Lemur catta (Ring-tailed lemur), Loris tardigradus (Slender loris), Microcebus murinus (Gray mouse lemur), Mirza zaza (Northern giant mouse lemur), Nycticebus coucang (Slow loris), Otolemur crassicaudatus (Greater galago), Perodicticus potto (Potto), Propithecus diadema (Diademed sifaka), Propithecus tattersalli (Golden-crowned sifaka), Varecia rubra (Red ruffed lemur).

#### Primates from Busch Gardens

The blood samples from chimpanzees (*Pan troglodytes*, *n* = 2), gorillas (*Gorilla*, *n* = 3)

Orangutan (*n* = 1, *Pongo pygmaeus*), red ruffed lemur (*n* = 1, *Varecia variegata*), and White-fronted marmoset (*n* = 1, *Callithrix geoffroyi*) were opportunistically collected and banked during routine health exams from these zoo-based animals located at Busch Gardens Tampa (Tampa, Florida).

#### Existing data from primates

The data from other monkeys are described in the companion papers for rhesus macaque (skin, blood, adipose, cerebral cortex, liver, lung, muscle [14]), vervet monkey (whole blood, prefrontal cortex and liver [13]), and common marmosets (*n* = 95 blood samples [15]).

#### Human tissue samples

We analyzed previously generated methylation data from *n* = 1352 human tissue samples (adipose, blood, bone marrow, dermis, epidermis, heart, keratinocytes, fibroblasts, kidney, liver, lung, lymph node, muscle, pituitary, skin, spleen) from individuals whose ages ranged from 0 to 101 years. Out of the 1352 tissues, *n* = 655 came from women (Supplementary Table S12).

The tissue samples came from four sources: tissue and organ samples from the National NeuroAIDS Tissue Consortium [73], Blood samples from the Cape Town Adolescent Antiretroviral Cohort study [74] and the PEG study [75], skin and other primary cells provided by Ken Raj [76]. Ethics approval (IRB#18-000315).

#### DNA extraction

DNA was extracted on an automated nucleic acid extraction platform Anaprep (Biochain) using a magnetic bead based extraction method and Tissue DNA Extraction Kit (AnaPrep).

#### DNA methylation data

The genome coordinates of each CpG probe on the mammalian array is provided on the Github page of the Mammalian Methylation Consortium [77] https://github.com/shorvath/MammalianMethylationConsortium/

The manifest file of the mammalian array can also be found on Gene Expression Omnibus (GPL28271). The SeSaMe normalization method was used to define beta values for each probe [78].

#### Penalized Regression models

Details on the clocks (CpGs, genome coordinates) and R software code are provided in the Supplement. Penalized regression models were created with glmnet [79]. We investigated models produced by both “elastic net” regression (alpha = 0.5). The optimal penalty parameters in all cases were determined automatically by using a tenfold internal cross-validation (cv.glmnet) on the training set. By definition, the alpha value for the elastic net regression was set to 0.5 (midpoint between Ridge and Lasso type regression) and was not optimized for model performance.

We performed a cross-validation scheme for arriving at unbiased (or at least less biased) estimates of the accuracy of the different DNAm based age estimators. One type consisted of leaving out a single sample (LOOCV) from the regression, predicting an age for that sample, and iterating over all samples. The other type, LOFO10, denotes the ten-fold cross-validation estimates of age where each fold contains the same proportion of primate species.

A critical step is the transformation of chronological age (the dependent variable). While no transformation was used for the pan-tissue clock for baboons, we used a log linear transformation for the dual species clock of absolute age. For the primate clock, we used the following transformation: sqrt(Age + 1), i.e. after adding an offset of 1 year, we formed the square root transformation. The square root transformation outperformed the log transformation and the identity transformation with respect to cross validation based estimates of accuracy.

We defined relative age as ratio: Relative age = Age/maxLifespan where the maximum lifespan for the different species was chosen from the anAge data base [19], e.g. the human maximum lifespan was determined to be 122.5. For the sake of reproducibility, we report the maximum lifespan and average age at sexual maturity in Supplementary Table S2.

#### Epigenome wide association studies (EWAS) of age in baboons

EWAS was performed in each tissue separately using the R function "standardScreeningNumericTrait" from the "WGCNA" R package. Next the results were combined across tissues using Stouffer's meta-analysis method.

#### EWAS of age across all primate species

Our meta analysis for EWAS of age in primate species combined correlation test statistics calculated in 29 different species-tissue strata with a minimal sample size of 10 (N ≥ 10). In the first stage, we combined the EWAS results across tissues within the same species to form species specific meta-EWAS results with one exception in lemurs. We analyzed *n* = 91 Strepsirrhini samples from both genders (68 blood and 23 skin tissues) from 25 distinct Strepsirrhini species. The low number of animals per Strepsirrhini species made it impossible to conduct EWAS of age in each species. Therefore, we pooled all Strepsirrhini samples into a single class as if they derived from a single lemur species. In the second stage, we combined species EWAS results to form a final meta-EWAS of age. All the meta analyses in both stages were performed by the unweighted Stouffer’s method.

### Human methylation array studies

#### Framingham Heart Study (FHS, *N* = 2356)

We used blood methylation data from 2,356 individuals composed of 888 pedigrees from the Framingham Heart cohort [80], a large-scale longitudinal study started in 1948, initially investigating risk factors for cardiovascular disease (CVD). The FHS cohort contains blood DNA methylation profiling at exam 8. A human epigenetic clock analysis of these data is presented in many articles including [11, 81].

#### Jackson Heart Study (JHS, *N* = 1747)

The JHS is a large, population-based observational study evaluating the etiology of cardiovascular, renal, and respiratory diseases among African Americans residing in the three counties (Hinds, Madison, and Rankin) that make up the Jackson, Mississippi metropolitan area. The age at enrollment for the unrelated cohort was 35–84 years; the family cohort included related individuals > 21 years old. JHS ancillary study ASN0104, available with both phenotype and DNA methylation array data.

#### ROSMAP (*N* = 700)

We analyzed previously generated DNA methylation data from Caucasian subjects from the Religious Order Study (ROS) and the Rush Memory and Aging Project (MAP) [82] Both are longitudinal community based cohort studies of aging and dementia. The majority of participants in both studies are 75–80 years old at baseline with no known dementia. All participants agree to organ donation at death. Participants sign and informed consent, repository consent, and Anatomical Gift Act.

### Supplementary Information

Below is the link to the electronic supplementary material.

Supplementary file1 (DOCX 4798 KB)

Supplementary file2 (XLSX 7485 KB)

## Data Availability

The methylation data pertaining to primates will be included in the forthcoming official data release from the Mammalian Methylation Array Consortium [50]. The methylation data for primates generated in this study are part from the data release from the Mammalian Methylation Array Consortium. The mammalian methylation array is available through the non-profit Epigenetic Clock Development Foundation (https://clockfoundation.org/).

## Code availability

Details on the clocks (CpGs, genome coordinates) and R software code are provided in Supplementary Note 1 and in Supplementary Table S4. Genome annotations of the CpGs can be found on Github https://github.com/shorvath/MammalianMethylationConsortium
